# Common SNPs in Myelin Transcription Factor 1-Like (*MYT1L*): Association with Major Depressive Disorder in the Chinese Han Population

**DOI:** 10.1371/journal.pone.0013662

**Published:** 2010-10-27

**Authors:** Ti Wang, Zhen Zeng, Tao Li, Jie Liu, Junyan Li, You Li, Qian Zhao, Zhiyun Wei, Yang Wang, Baojie Li, Guoyin Feng, Lin He, Yongyong Shi

**Affiliations:** 1 Bio-X Center and Affiliated Changning Mental Health Center, Key Laboratory for the Genetics of Developmental and Neuropsychiatric Disorders, Ministry of Education, Shanghai Jiao Tong University, Shanghai, People's Republic of China; 2 Institutes for Nutritional Sciences, Shanghai Institute of Biological Sciences, Chinese Academy of Sciences, Shanghai, People's Republic of China; 3 Shanghai Institute of Mental Health, Shanghai, People's Republic of China; 4 Institutes of Biomedical Sciences, Fudan University, Shanghai, People's Republic of China; University of Muenster, Germany

## Abstract

**Background:**

Myelin transcription factor 1-like (*MYT1L*) is a member of the myelin transcription factor 1 (*MYT1*) gene family, and the neural specific, zinc-finger-containing, DNA-binding protein that it encodes plays a role in the development of the nervous system. On the basis of a recent copy number variation (CNV) study showing that this gene is disrupted in mental disorder patients, we investigated whether *MYT1L* also plays a role in MDD.

**Methods:**

In this study, 8 SNPs were analyzed in 1139 MDD patients and 1140 controls of Chinese Han origin.

**Results:**

Statistically significant differences were noted between cases and controls for rs3748989 (allele: permutated p = 0.0079, corrected p = 0.0048, genotype: corrected p = 0.0204). A haplotype of rs1617213 and rs6759709 G-C was also significant (permutated p = 0.00007).

**Conclusion:**

Our results indicate that *MYT1L* may be a potential risk gene for MDD in the Chinese Han population.

## Introduction

Major depressive disorder (MDD) is a common and severe mental disorder with a prevalence of at least 10% in the general population [1]–[2]. Although twin studies indicate only 40–50% heritability in MDD, adoption studies indicate the role of genetic factors [3]. Many candidate genes, such as *SLC6A4* and *5HTR2A*, have been associated with MDD in recent decades [2]. A number of genes have also been identified as being common to several psychotic disorders such as schizophrenia (SCZ) and bipolar disorder (BP) [4]. The classic example is *DISC1*, which is linked to both SCZ and MDD [5].

The gene myelin transcription factor 1-like (*MYT1L*, also known as *NZF1*) is located on chromosome 2p25.3. The gene spans 542081 bp, and has 25 exons. Exon 1 to exon 5 and the distal part of exon 25 are untranslated regions (UTRs), while the other 19 exons and the proximal part of exon 25 are coding regions. *MYT1L* is a member of the myelin transcription factor 1 (*MYT1*) gene family. These genes encode a family of neural specific, zinc-finger-containing DNA-binding proteins, which play a role in the development of the nervous system. In a recent copy number variation (CNV) study, *MYT1L* along with 3 other candidate genes was found to be disrupted in SCZ patients [6].

In this case-control study, we sought to determine whether *MYT1L* is associated with MDD.

## Materials and Methods

### 1 Ethics Statement

The complete details of the entire study design and procedures involved were submitted to the ethics committee of Bio-X center Shanghai Jiaotong University. After approval was obtained, we initiated this research in accordance with the defined protocols. Each participant was clearly explained about the procedure and purpose of this study. Written informed consent for this genetic study was given by each participant. All data were recorded anonymously but if the participants requested to withdraw their file, the data were destroyed.

### 2 Subjects

The patients and controls recruited in this study were biologically unrelated and all were of the Han Chinese ethnicity and belonged to the local Shanghai population. We enrolled 1139 patients with major depressive disorder (487 male patients and 652 female patients). All patients were treated at the same clinic, and the two-year case history of each patient was analyzed to ensure that he/she did not have a bipolar tendency. In this study, all the patients recruited had severe depressive symptoms. All patients were assessed by using the Structured Clinical Interview for DSM disorders. All the patients had a sole diagnosis of depression, and some had additional features of anxiety. We included only patients with Hamilton Depression Rating Scale (HAMD) scores greater than 20. Cases with high HAMD scores could be thought of as a subtype of depressive disorder. The control group consisted of 1140 randomly selected normal individuals (374 males and 766 females). The average age of onset of MDD was 35.1 (±11.6) years, and the average age of the controls was 58.7 (±9.9) years. All the patients were interviewed by 2 independent psychiatrists and were diagnosed strictly according to the DSM-IV criteria. Patients who had organic disorders were excluded from the study. Controls were recruited from among volunteers randomly selected from local Shanghai communities. All the controls gave informed consent and were local citizens with both their parents belonging to Shanghai and no family history of psychiatric disorders. Written informed consent for this genetic study was provided by each participant. The demographic characteristics of the study population are listed in table 1.

**Table 1:** Sociodemographic characteristics of the sample population.

Study sample	Sample Size	Mean Age (SD)	Mean Age of Onset (SD)	Male/Female	Female %
MDD	1,139	36.2(12.8)	35.1(11.6)	484/655	57.2
Control	1,140	58.7(9.9)		349/791	67.2

MDD: major depressive disorder; SD: standard deviation.

### 3 Genotyping

**Figure 1:**
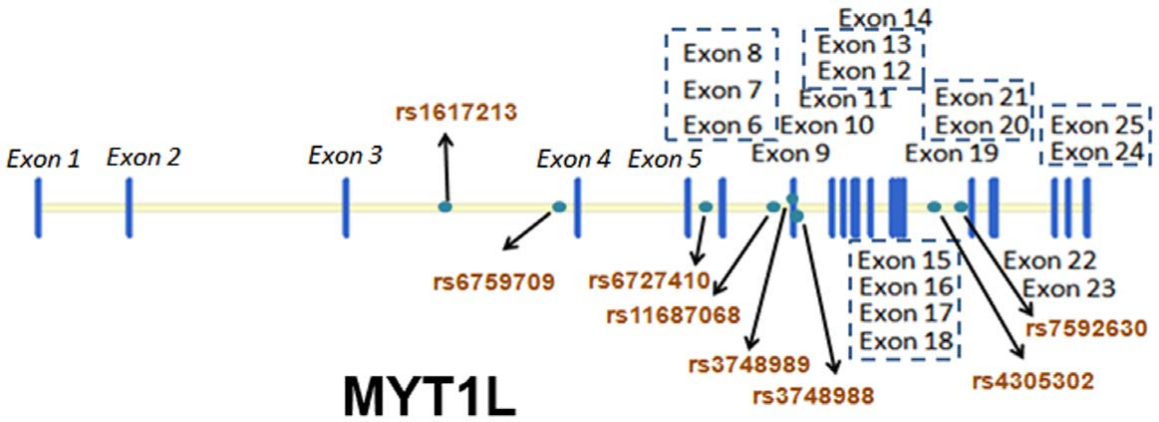
Exons of *MYT1L*, and eight tag SNPs we selected. This figure was created by software Vector NTI. The blue strips represent all the 25 exons of *MYT1L*. Since the whole length of gene *MYT1L* is 542081 bp and most exons are shorter than 400 bp and are closely located, the 25 exons seem very closely located and crowded. The exons located by the same strip, such as exons 6 to 8, exons 12 to 13, exons 15 to 18, and exons 24 to 25, have been enclosed in a dot line square. Exon 1 to exon 5 and the distal part of exon 25 are untranslated regions (UTRs), while the other 19 exons and the proximal part of exon 25 are coding regions. Noncoding exons are showed in italics. The green dots identify the 8 SNPs selected from *MYT1L*, and the arrow head points to their rs numbers. Markers rs3748988 and rs3748989 are located in exon 9, and the other 6 are located in introns. Exon 9 is only 353-bp long and encodes a polypeptide of 119 amino acids.

There are 25 exons in *MYT1L*. Exon 1 to exon 5 and the distal part of exon 25 are untranslated regions (UTRs), while the other 19 exons and the proximal part of exon 25 are coding regions. We focused on the region extending from exon 6 to exon 24. We selected tag SNPs using the software Haploview 4.1, with minor allele frequency (MAF) ≥0.2 and r^2^≥0.5 in the Han Chinese population in Beijing. Due to the non-availability of allele frequency data of many markers for the CHB population, we only found 2 in coding regions (exon 9). Then we selected others with a view to obtaining uniform coverage of the coding regions. Finally, we chose 8 tag SNPs (rs1617213, rs6759709, rs6727410, rs11687068, rs3748989, rs3748988, rs4305302, and rs7592630) for genotyping. These SNPs span the region where most exons are located. Except for rs3748989 and rs3748988, which are located in exon 9, the markers are all intronic SNPs (the location of these SNPs and exons of *MYT1L* are displayed in Fig. 1). We tested our SNP variability by using a web tool provided by the Broad Institute http://www.broadinstitute.org/mpg/tagger/server.html [7]. On performing this test with r^2^≥0.5 and MAF ≥0.2, our markers could capture 54% of the variability in the whole region. We performed genotyping using the ABI 7900 DNA detection system (Applied Biosystems, Foster City, California) with TaqMan® probes supplied by Applied Biosystems. Every assay was tested twice in 32 samples during the preliminary experiments. The results of these duplicate samples were completely consistent (100%). The standard 5-µl polymerase chain reaction (PCR) reaction was carried out using TaqMan® Universal PCR Master Mix reagent kits according to the protocol recommended by the manufacturer.

### 4 Statistical analysis

Odds ratios, allele frequency, and genotypic association were calculated by using http://analysis.bio-x.cn [Shi and He 2005]. This is a user friendly platform that integrates analysis tools especially suited to association studies. We used Haploview 4.1 [8] to estimate the Hardy–Weinberg equilibrium, linkage disequilibrium, and allelic and haplotype distribution. For precise results, p values of the alleles were permutated 100,000 times. To reduce the type I error rate, we also ran the program SNPSpD [9], which reflects the linkage disequilibrium among markers and correct p values.

## Results

We genotyped 8 SNPs on all the samples, and found that deviation from the Hardy–Weinberg equilibrium was absent for all cases and controls. MDD patients and controls showed statistically significant differences for 3 SNPs in the allelic and genotypic distribution (rs3748989, p = 0.0008 for allele, p = 0.0034 for genotype; rs3748988, p = 0.024 for allele, p = 0.0051 for genotype; rs7592630, p = 0.0369 for genotype). The results are shown in table 2 and Supporting information –Table S1. Even after permutation on Haploview and correction by SNPSpD (the effective number of tests is 6), rs3748989 continued to be significantly associated with MDD (for allele, permutated p = 0.0079, corrected p = 0.0048; for genotype, corrected p = 0.0204), and the genotypic frequency of rs3748988 still remained significant (for genotype, corrected p = 0.0306). Power analysis was performed by using the software GPower. The power was 0.9550452, with effect size d = 0.14 [d = ln(or)/1.65], α = 0.05 (odd's ratio was for rs3748989).

**Table 2:** Association analysis of 8 SNPs in the MYT1L gene among MDD patients and controls.

SNP ID		MAF	Allele	Frequency	Call Rates	Odds Ratio	p value	Permutated p value	Pearson's p value
			A	G					
rs1617213	MDD	0.256	1731(0.7818)	483(0.2182)	0.97	0.873	0.0706	0.4777	0.0974
Intron	Control		1798(0.8041)	438(0.1959)	0.98				
			C	T					
rs6759709	MDD	0.256	479(0.2228)	1671(0.7772)	0.94	1.1273	0.1031	0.6139	0.2511
Intron	Control		446(0.2027)	1754(0.7973)	0.96				
			A	G					
rs6727410	MDD	0.411	838(0.3813)	1360(0.6187)	0.96	0.9264	0.2456	0.9105	0.4616
Intron	Control		886(0.3995)	1332(0.6005)	0.97				
			A	G					
rs11687068	MDD	0.356	1457(0.6599)	751(0.3401)	0.97	0.924	0.1946	0.8446	0.2412
Intron	Control		1348(0.6774)	642(0.3226)	0.87				
			C	T					
**rs3748989**	MDD	0.222	1617(0.7707)	481(0.2293)	0.92	1.2653	**0.0008**	**0.0079**	**0.0034**
Exon 9	Control		1626(0.7265)	612(0.2735)	0.98				
			A	G					
**rs3748988**	MDD	0.411	970(0.4385)	1242(0.5615)	0.97	1.1461	**0.024**	0.2019	**0.0051**
Exon 9	Control		907(0.4053)	1331(0.5947)	0.98				
			C	T					
rs4305302	MDD	0.359	754(0.3481)	1412(0.6519)	0.95	0.984	0.7845	1.0000	0.114
Intron	Control		769(0.3518)	1417(0.6482)	0.96				
			C	T					
rs7592630	MDD	0.411	1312(0.6102)	838(0.3898)	0.94	0.9136	0.1817	0.8222	**0.0369**
Intron	Control		1359(0.6315)	793(0.3685)	0.94				

MDD  =  major depressive disorder; SNP  =  single nucleotide polymorphism.

Odds ratio using SHEsis; p value using Haploview 4.1 and was permutated 100,000 times; Pearson's p value using SHEsis is for genotype frequency.

Since there is a difference in the proportion of males and females in our samples, we analyzed our data by using the PLINK software with age and gender as covariables. After correction, the p value was still smaller than 0.05. We also performed an association analysis stratified by gender in Haploview 4.1. The p values are still smaller than 0.05 in both gender groups.

**Figure 2:**
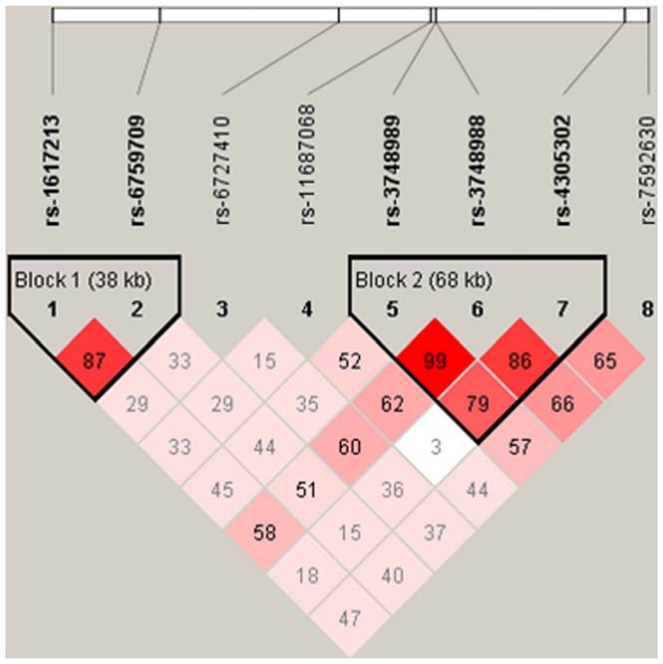
D' value for the 8 single nucleotide polymorphisms (SNPs). D' value between marker pairs is indicated by the shaded matrices. This structure was created by Haploview 4.1. There were 2 linkage-disequilibrium blocks in the selected region. The SNPs rs1617213 and rs6759709 were present in block 1. The SNPs rs3748989, rs3748988, and rs4305302 were present in block 2.

**Figure 3:**
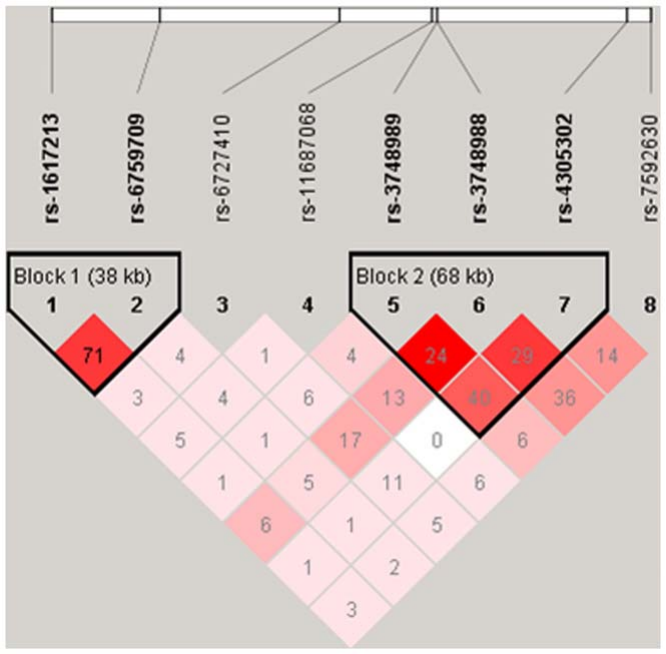
r^2^ value for 8 single nucleotide polymorphisms (SNPs). r^2^ value between the marker pairs is indicated by the shaded matrices. This structure was created by Haploview 4.1. There were 2 linkage-disequilibrium blocks in the selected region. The SNPs rs1617213 and rs6759709 were present in block 1. The SNPs rs3748989, rs3748988, and rs4305302 were in block 2. The SNPs rs1617213 is not correlated with either rs3748989 or rs3748988, since the r^2^ values were only 0.018 and 0.064, respectively. r^2^ values of rs3748989 and rs3748988 is 0.247.

The results of linkage disequilibrium analysis for these SNPs are shown in Figs. 2 and 3. Block 1 and block 2 were in linkage disequilibrium, with D'>0.6. Subsequent haplotype analysis for these 2 blocks showed a notable association in block 1 (rs1617213 and rs6759709 G-C; permutated p = 0.00007).

## Discussion

In our study, we found 1 SNP in *MYT1L* to be associated with MDD. The expression of the protein MYT1L, which is abundant in the brain, has never been studied for allelic association in any psychiatric disorder. However, in a genome-wide screen for CNVs with SCZ patients, Vrijenhoek *et al*. identified 2 duplications—*MYT1L* in the chromosomal region 2p25.3 and *CTNND2* at 5p15.2. Both these genes were disrupted at their terminal portions by breakpoints of 2 duplications [6]. Two other candidate genes implicated in their study were *NRXN1* and *ASTN2*. Each of these genes showed 1 deletion each. *NRXN1* has already been confirmed to be associated with SCZ. Although no definite relationship between the other 3 genes and mental disorders has yet been established, it is known that all these genes play important roles in neuronal function. CTNND2 catenin (cadherin-associated protein), delta 2 (neural plakophilin-related arm-repeat protein) is a member of the beta-catenin superfamily, which is abundantly present in neural tissue and is known to modulate neurite outgrowth and synaptic activity[10]. Deletion of *ASTN2* has been associated with SCZ, and CNV of this gene was recently found in patients with autism spectrum disorders (ASD) [11]. We suggest that the MYT1L gene may also confer risk for MDD.

*MYT1L* is highly homologous to myelin transcription factor 1 (*MYT1*); the former maps to human chromosome 2, while the latter maps to chromosome 20. Protein MYT1 is a representative of the Cys-Cys-His-Cys (CCHC) zinc-finger protein family, which has been highly conserved during evolution between both species and family members. MYT3 is the third member of this family [12]. Both MYT1 and MYT1L are widely expressed in developing neuronal cells. MYT1L can only be detected in neurons at the early stages of differentiation, but not in the glial lineage. This suggests that MYT1L has a role in the development of the central nervous system [13]–[14]. MYT1/MYT1L binding can result in the recruitment of histone deacetylases (HDACs) to target gene promoters, which results in transcriptional repression [15]; the resultant loss of MYT1 function may be compensated by MYT1L activity [16]. Although MDD in general is not classically considered to be a neurodevelopmental in origin, severe MDD has pervasive features that present in neurodevelopmental disorders. The neurodevelopmental hypothesis of SCZ was established 30 years ago and has been considered controversial for a long time; this hypothesis postulated that pre- and perinatally acting cerebral noxae caused disturbances of corticogenesis in the developing neuronal fiber systems, which are responsible for the later onset of the disease [17]. According to this hypothesis, an unidentified event occurring in utero might disturb the normal maturation of neuronal connections in SCZ patients [18]. David *et al.* found an altered plasticity of the hippocampal structure in a large proportion of the brains of SCZ patients [19]. Synaptic rearrangement and plasticity were found to be related to the expression of the embryonic form of neural cell adhesion molecule (NCAM). David *et al.* also reported that these changes were caused by the decrease in the levels of polysialylated NCAM. NCAM isoforms in the brain were reported to be critical for many neurodevelopmental processes, including neurulation, axonal outgrowth, and the establishment of neuronal connectivity [19]. All these findings suggest that abnormalities and disturbances in the development processes of the central nervous system might lead to the appearance of mental disorders. Since the protein MYT1L has been reported to play a role in the development of the central nervous system, we think this protein may also play a similar role in the pathogenesis of MDD.

This study represents the first attempt to test the association between *MYT1L* and MDD. We recruited 1139 patients and 1140 controls and used 8 SNPs as tag markers to investigate the role *MYT1L* in MDD patients of Chinese Han origin. The single marker, rs3748989, located in exon 9 was found to be strongly associated with MDD. Although this is a synonymous SNP, a different allele may influence the efficiency of transcription, the structural stability of transcripts, and the splicing signals [20], which result in differences in its expression. We have submitted the sequence of exon 9 with different allele in rs3748989 to RNAfold (http://rna.tbi.univie.ac.at//cgi-bin/RNAfold.cgi) for testing. There is an obvious difference in the minimum free energy between allele A (−43.53 kcal/mol) and allele G (−59.63 kcal/mol) of rs3748989. We think this difference may have an impact on their secondary RNA structures during transcription. A haplotype for rs1617213 and rs6759709 G-C was also significant, but the significance of this remains unclear. In this study, we only genotyped 8 markers; this is an obvious limitation of the study. We suggest that more markers should be genotyped in subsequent studies for a better mapping.

In summary, our results indicate that rs3748989 is probably associated with MDD and that *MYT1L* may be a potential risk gene for MDD in the Chinese Han population. To clarify the relation between this gene and MDD, further studies with more markers and cellular or protein expression experiments are needed.

## Supporting Information

Table S1 Details in association analysis of 8 SNPs in the MYT1L gene among MDD patients and controls.
(0.06 MB DOC)
