# Supplementary material for: Common SNPs in Myelin Transcription Factor 1-Like (MYT1L): Association with Major Depressive Disorder in the Chinese Han Population
Source: PLoS One. 2010 Oct 27;5(10):e13662. doi: 10.1371/journal.pone.0013662 (PMC2965102; doi:10.1371/journal.pone.0013662)
Supplement: Table S1 — Details in association analysis of 8 SNPs in the MYT1L gene among MDD patients and controls. (0.06 MB DOC) [file pone.0013662.s001.doc]

**Table S1 Details in association analysis of 8 SNPs in the MYT1L gene among MDD patients and controls**

| **SNP ID** | | **MAF** | **Allele Frequency** | | **Call Rates** | **Odds Ratio** | **p value** | **Permutated p value** | **Genotype Frequency** | | | **Pearson's p value** | **H-W p value** |  |
| --- | --- | --- | --- | --- | --- | --- | --- | --- | --- | --- | --- | --- | --- | --- |
|  |  |  | A | G |  |  |  |  | AA | GG | AG |  |  |  |
| rs1617213 | MDD | 0.256 | 1731(0.7818) | 483(0.2182) | 0.97 | 0.873 | 0.0706 | 0.4777 | 673(0.6079) | 49(0.0443) | 385(0.3478) | 0.0974 | 0.9523 |  |
| Intron | Control |  | 1798(0.8041) | 438(0.1959) | 0.98 |  |  |  | 728(0.6512) | 48(0.0429) | 342(0.3059) |  |  |  |
|  |  |  | C | T |  |  |  |  | CC | TT | CT |  |  |  |
| rs6759709 | MDD | 0.256 | 479(0.2228) | 1671(0.7772) | 0.94 | 1.1273 | 0.1031 | 0.6139 | 55(0.0512) | 651(0.6056) | 369(0.3433) | 0.2511 | 0.4374 |  |
| Intron | Control |  | 446(0.2027) | 1754(0.7973) | 0.96 |  |  |  | 50(0.0455) | 704(0.6400) | 346(0.3145) |  |  |  |
|  |  |  | A | G |  |  |  |  | AA | GG | AG |  |  |  |
| rs6727410 | MDD | 0.411 | 838(0.3813) | 1360(0.6187) | 0.96 | 0.9264 | 0.2456 | 0.9105 | 168(0.1529) | 429(0.3904) | 502(0.4568) | 0.4616 | 0.2575 |  |
| Intron | Control |  | 886(0.3995) | 1332(0.6005) | 0.97 |  |  |  | 183(0.1650) | 406(0.3661) | 520(0.4689) |  |  |  |
|  |  |  | A | G |  |  |  |  | AA | GG | AG |  |  |  |
| rs11687068 | MDD | 0.356 | 1457(0.6599) | 751(0.3401) | 0.97 | 0.924 | 0.1946 | 0.8446 | 473(0.4284) | 120(0.1087) | 511(0.4629) | 0.2412 | 0.857 |  |
| Intron | Control |  | 1348(0.6774) | 642(0.3226) | 0.87 |  |  |  | 461(0.4633) | 108(0.1085) | 426(0.4281) |  |  |  |
|  |  |  | C | T |  |  |  |  | CC | TT | CT |  |  |  |
| **rs3748989** | MDD | 0.222 | 1617(0.7707) | 481(0.2293) | 0.92 | 1.2653 | **0.0008** | **0.0079** | 623(0.5939) | 55(0.0524) | 371(0.3537) | **0.0034** | 0.4393 |  |
| Exon 9 | Control |  | 1626(0.7265) | 612(0.2735) | 0.98 |  |  |  | 597(0.5335) | 90(0.0804) | 432(0.3861) |  |  |  |
|  |  |  | A | G |  |  |  |  | AA | GG | AG |  |  |  |
| **rs3748988** | MDD | 0.411 | 970(0.4385) | 1242(0.5615) | 0.97 | 1.1461 | **0.024** | 0.2019 | 194(0.1754) | 330(0.2984) | 582(0.5262) | **0.0051** | 0.3872 |  |
| Exon 9 | Control |  | 907(0.4053) | 1331(0.5947) | 0.98 |  |  |  | 192(0.1716) | 404(0.3610) | 523(0.4674) |  |  |  |
|  |  |  | C | T |  |  |  |  | CC | TT | CT |  |  |  |
| rs4305302 | MDD | 0.359 | 754(0.3481) | 1412(0.6519) | 0.95 | 0.984 | 0.7845 | 1.0000 | 121(0.1117) | 450(0.4155) | 512(0.4728) | 0.114 | 0.9738 |  |
| Intron | Control |  | 769(0.3518) | 1417(0.6482) | 0.96 |  |  |  | 147(0.1345) | 471(0.4309) | 475(0.4346) |  |  |  |
|  |  |  | C | T |  |  |  |  | CC | TT | CT |  |  |  |
| rs7592630 | MDD | 0.411 | 1312(0.6102) | 838(0.3898) | 0.94 | 0.9136 | 0.1817 | 0.8222 | 392(0.3647) | 155(0.1442) | 528(0.4912) | **0.0369** | 0.6068 |  |
| Intron | Control |  | 1359(0.6315) | 793(0.3685) | 0.94 |  |  |  | 444(0.4126) | 161(0.1496) | 471(0.4377) |  |  |  |
| MDD = major depressive disorder; SNP = single nucleotide polymorphism. | | | | | | | | | | | | | | |
| Odds ratio using SHEsis; p value using Haploview 4.1 and was permutated 100,000 times; Pearson’s p value using SHEsis | | | | | | | | | | | | | | |
